# The Medical Training of Missionaries

**Published:** 1921-03-19

**Authors:** 


### LIVINGSTONE COLLEGE AFTER THE WAR.

### The Medical Training of Missionaries.

It is interesting to see the first report available since Livingstone College, Leyton, E., ceased to be a war hospital and reverted to its proper purpose of training medical missionaries. The Principal, Dr. Loftus E. Wigram, and his Vice-principal, Dr. T. Jays, now have to deliver most of the lectures as the staff has not been fully recruited since the war. But these, being both resident, are always available, and valuable honorary help continues to be given by distinguished physicians in their special subjects. Clinical teaching is also given at St. James the Less Medical Mission, Bethnal Green; and surgical experience can be obtained by the arrangements made with Poplar Hospital for Accidents. Other students attend weekly at the Forest Lodge Convalescent Home, and the out-patient department of Walthamstow Hospital. There are also other opportunities for clinical experience, and the wards of the Seamen's Hospital have been visited in connection with the Vice-principal's lectures on Tropical diseases. Students have averaged seventeen for each term, and seven, all of whom were residents, for the full session. The places to which they go after completing their studies range from Papua to Africa. Missionaries come from their missions on leave for the benefit of the course. Twenty-nine students represent seven nationalities, so the College is educative in more senses than one. The College is financed by fees and by annual subscriptions, but though the former have been increased by fifty per cent. on pre-war rates, prices have risen in a higher proportion, and last year a deficit of £360 was incurred. In the summer it was decided to admit women students, and of several applications received two women took the course. This number will almost certainly grow, and it will be no longer necessary for the Principal to refer women applicants to the Bermondsey, Islington, and Mildmay Medical Mission. On Commemoration Day, three former students spoke of their experiences in the Mission field, and agreed on the necessity of medical training for all missionaries before going abroad. This knowledge is not only useful to the people among whom a missionary works, but is often the means of saving his own life by equipping him to deal with all preventable causes of illness. Next year a residential vacation course will be held during July, when lectures on the care of health in the Tropics, together with practical clinical work, will be provided. The income of the College is sufficient to prove that it is still too little known, and we are afraid that many supporters of foreign missions are still unaware of the necessity for medical training to all missionaries in foreign lands. A visit to the College, to which Hoe Street on the G.E.R. is the nearest station, would prove a useful eye-opener to many, who would come away with a deeper understanding of the value of medical science in this department of the Mission field.

